# Cardiac events and dynamic echocardiographic and electrocardiogram changes following osimertinib treatment in lung cancer

**DOI:** 10.3389/fcvm.2024.1485033

**Published:** 2024-12-16

**Authors:** Jonathan N. Le, Jordan O. Gasho, Olivia Peony, Asneh Singh, Katrina D. Silos, Sungjin Kim, Anthony T. Nguyen, Mitchell Kamrava, Amin Mirhadi, Behrooz Hakimian, Karen L. Reckamp, Kamya Sankar, Raymond H. Mak, Andriana P. Nikolova, Katelyn M. Atkins

**Affiliations:** ^1^ Department of Medicine, Cedar-Sinai Medical Center, Los Angeles, CA, United States; ^2^ Department of Radiation Oncology, Cedars-Sinai Medical Center, Los Angeles, CA, United States; ^3^ Biostatistics Research Center, Samuel Oschin Comprehensive Cancer Center, Cedars-Sinai Medical Center, Los Angeles, CA, United States; ^4^ Division of Medical Oncology, Cedars-Sinai Medical Center, Los Angeles, CA, United States; ^5^ Department of Radiation Oncology, Brigham and Women’s Hospital/Dana-Farber Cancer Institute, Boston, MA, United States; ^6^ Department of Cardiology, Smidt Heart Institute, Cedars-Sinai Medical Center, Los Angeles, CA, United States

**Keywords:** osimertinib, lung cancer, cardiac toxicity, echocardiogram, EGFR

## Abstract

Osimertinib is first-line treatment for epidermal growth factor (EGFR)-mutated non-small cell lung cancer (NSCLC) and has been associated with cardiotoxicity. However, the nature of cardiac remodeling and associated risk factors remains incompletely understood. Retrospective analysis of NSCLC patients with ≥1 echocardiogram post-osimertinib between 2007 and 2022 was performed. The cumulative incidence of grade ≥2 cardiac common terminology criteria for adverse events (CTCAE) was estimated and Fine and Gray regressions performed (non-cardiac death as competing risk). Eighty-five patients [mean [interquartile range, IQR], 68 [60–75] years; 67% female; 12% with pre-existing heart conditions] met inclusion criteria. With a median follow up of 34.7 months, the 2-year cumulative incidence of grade ≥2 and grade ≥3 cardiac events were 19.2% and 8.5%, respectively. There was an increased risk of grade ≥2 cardiac CTCAE with pre-existing arrhythmia [hazard ratio(HR) 3.90, 95%CI, 1.11–13.72; *p* = 0.034] and higher body mass index (HR 1.07, 95%CI, 1.00–1.14; *p* = 0.04). Following osimertinib (vs. baseline), the median QTc was prolonged (451 vs. 437 ms; *p* < 0.001) and LVEF ≤50% was more common (10.6% vs. 5.3%; *p* = .046). Osimertinib treatment was associated with QTc prolongation and reduced LVEF. BMI was identified as a potentially modifiable risk factor for osimertinib-associated cardiotoxicity, worthy of further study.

## Introduction

Lung cancer mortality has been declining over the past two decades driven in part by advances in systemic therapies (1–3). However, lung cancer survivors face increasing risk of cardiovascular disease (CVD) from both pre-existing risk factors as well as excess risk from cancer therapies. Patients with lung cancer have the highest prevalence of concomitant CVD compared to patients with other malignancies, with more than 40% harboring pre-existing CVD (4). Among lung cancer survivors, CVD is the leading cause of death (5). Further, a number of epidemiologic studies have demonstrated shared risk factors between lung cancer and CVD (4, 6, 7). In addition to these baseline and shared risk factors, lung cancer treatment often includes multi-modality treatment—radiotherapy, cytotoxic chemotherapy, immune checkpoint inhibitors, and/or tyrosine kinase inhibitors—for which treatment-associated cardiac toxicities are observed and associated with pre-existing cardiovascular co-morbidities (8).

For epidermal growth factor (EGFR)-mutated non-small cell lung cancer (NSCLC), osimertinib is an oral, third-generation EGFR tyrosine kinase inhibitor (EGFR-TKI) that selectively inhibits EGFR mutations and T790M resistance mutations, and is the first-line systemic therapy for advanced disease and as adjuvant therapy in patients with resected disease (2, 9, 10). While osimertinib has been shown to improve survival outcomes compared to other systemic therapies (earlier generation TKIs such as erlotinib or gefitinib, or platinum therapy plus pemetrexed, respectively) (9, 11), it has been associated with higher rates of cardiotoxicity compared to earlier generation EGFR-TKIs (9, 12, 13). Indeed, a recent pharmacovigilance study using the FDA Adverse Events Reporting System (FAERS), reported that osimertinib (compared to standard therapies) was associated with higher rates of grade 3 or greater cardiac events such as QTc prolongation, cardiac failure, a decline in left ventricular ejection fraction (LVEF), among others (14). However, given the voluntary nature of the reporting and limited toxicity details, it may not accurately reflect the true incidence and extent of toxicity and additional studies characterizing osimertinib-associated cardiac events are warranted.

Together, the above studies highlight the significance of osimertinib-associated cardiac toxicity and underscore the need for more nuanced understanding of the cardiac remodeling that occurs with such therapy. To this end, we developed a single institution, retrospective cohort with detailed echocardiographic and electrocardiographic profiling in patients treated with osimertinib to investigate rates of cardiovascular toxicity and to identify predictive factors associated with cardiac events and mortality.

## Methods

### Study design

This was a retrospective analysis of patients with NSCLC treated with osimertinib between 2007 and 2022 at Cedar-Sinai Medical Center (Los Angeles, California). Patients were identified using a natural language search engine, DEEP-6 AI (Pasadena, California), with key terms “lung cancer”, “osimertinib”, and “echocardiogram.” Eligible patients included those with any stage NSCLC treated with osimertinib with at least one echocardiogram available after the initiation of osimertinib (Supplementary Figure 1). This study complied with the prinicples of the Declaration of Helsinki and was approved by the Cedars-Sinai Medical Center institutional review board with a waiver of informed consent.

### Cardiovascular events and follow-up

An in-depth manual review of the electronic medical record including past medical history, notes (consultation, follow-up, emergency department visits, and admissions), and diagnostic/imaging reports was used to identify cardiac events. Cardiovascular adverse events were defined according to the National Cancer Institute (NCI) Common Terminology Criteria for Adverse Events (CTCAE) version 5.0 (15). Event types/groups included heart failure, left ventricular ejection fraction (LVEF) reduction, myocardial infarction, arrhythmia (e.g., supraventricular tachycardia, atrial fibrillation, etc), QTc prolongation, valvular disease, pericardial disease. By CTCAE, grade 2 generally means non-urgent medical intervention required, grade 3 is symptomatic and/or urgent intervention required (i.e., hospitalization), grade 4 is life-threatening consequences or urgent intervention required, while grade 5 is death.

Twelve-lead electrocardiogram (ECG) and echocardiogram data were extracted from patients within 3 years prior to osimertinib administration and at any time after osimertinib was started. In patients with multiple ECGs and echocardiograms, the most recent study before and after osimertinib administration was used for analysis. Echocardiography studies were performed and interpreted by a multitude of observers and the original diagnostic reports were utilized for analysis and not re-interpreted for this study. QTc prolongation, calculated by Bazett formula (16), was defined as >445 ms in men and >460 ms in women. Patients were classified to have left ventricular hypertrophy by ECG if they met either the modified Cornell or Sokolow-Lyon criteria (17, 18). The endpoints of grade ≥2 (or grade ≥3) cardiac events and all-cause mortality were defined as time from the start of osimertinib to the date of the first cardiac CTCAE, or death, whichever occurred first.

### Statistical analysis

Comparisons between patients with and without cardiac events were made using analysis of variance or Wilcoxon rank-sum test for continuous variables and chi-square test or Fisher's exact test for categorical variables as appropriate. Data are presented as number of patients (%) for categorical variables and mean [± standard deviation (SD)] or median [interquartile range (IQR)] for continuous variables. Median follow-up was calculated using the reverse Kaplan-Meier method (19). Cumulative incidence estimates of cardiac events were estimated using non-cardiac death as a competing risk. Cumulative incidence estimates of all-cause mortality was calculated as 1—Kaplan Meier method (20). Univariate and multivariable analyses of cardiac events and all-cause mortality were carried out using a Fine-Gray proportional subdistribution hazards regression model (21) and a Cox proportional hazards model (22), respectively. Model assumptions were assessed by examining interaction effects between variables of interest and functions of time and scaled Schoenfeld residuals (23). Multivariable analyses were performed using a stepwise variable selection procedure based on Akaike Information Criterion (24). In the multivariable analysis, multicollinearity was assessed using tolerance and the variance inflation factor. Since hypothesis testing of unique baseline and post-Osimertinib ECG and echocardiogram parameters was limited to simple comparative analysis in this exploratory study, multiple testing correction was not deemed necessary. Analyses were performed using R package version 4.3.0 with two-sided tests at a significance level of 0.05 (25).

## Results

### Clinical characteristics

Of approximately 500 patients assessed for study eligibility, 85 met inclusion criteria (Supplementary Figure 1). The median age was 68 years [interquartile range (IQR), 60–75 years], 67.1% patients were female (Table 1). The cohort was comprised of 64.7% white and 15.3% Hispanic/Latin(x) individuals. In total, at baseline 49.4% of patients had hypertension, 36.5% with hyperlipidemia, 40.5% with history of tobacco use (only *n* = 2 current smokers), 16.5% with diabetes mellitus, and 11.8% with coronary heart disease. Most patients (82.1%) had metastatic (Stage IV) disease at the time of starting osimertinib. Osimertinib was used as first-line therapy in 36.2% of patients, as our cohort was treated in eras both prior to and after approval of osimertinib as first line therapy. Most patients (54.9%) were treated with cytotoxic chemotherapy, 25.3% with other EGFR inhibitors, 10.5% with immune checkpoint inhibitors, and 32.5% with thoracic radiotherapy.

**Table 1:** Baseline patient characteristics stratified by cardiac event status.

	All patients (*N* = 85)	No cardiac events (*n* = 68)	Cardiac events (*n* = 17)	*P*-value
Age, years	67.7 (± 10.8)	67.1 (±10.1)	70 (±13.4)	0.329
Body mass index (kg/m^2^)	25.1 (21.0–27.5)	24.8 (20.8–27.0)	25.9 (23.7–31.2)	0.170
Sex				
Female	57 (67.1)	46 (67.7)	11 (64.7)	0.817
Male	28 (32.9)	22 (32.4)	6 (35.3)	
Ethnicity				
Hispanic or Latin(x)	13 (15.3)	10 (14.7)	3 (17.7)	0.349
Non-Hispanic or Latin(x)	70 (82.4)	57 (83.8)	13 (76.5)	
Unknown	2 (2.4)	1 (1.5)	1 (5.9)	
Race				
Asian	20 (23.5)	16 (23.5)	4 (23.53	0.074
Black or African American	2 (2.4)	1 (1.5)	1 (5.9)	
Hawaiian/Pacific Islander	1 (1.2)	0 (0)	1 (5.9)	
Other	5 (5.9)	3 (4.4)	2 (11.8)	
Unknown	2 (2.4)	1 (1.5)	1 (5.9)	
White	55 (64.7)	47 (69.1)	8 (47.1)	
Smoking status				
Ever smoker	34 (40.5)	28 (41.8)	6 (35.3)	0.626
Non-smoker	50 (59.5)	39 (58.2)	11 (64.7)	
Medical history				
Any coronary heart disease	10 (11.8)	8 (11.8)	2 (11.8)	1.000
HFrEF	1 (1.2)	0 (0)	1 (5.9)	0.200
Chronic kidney disease	2 (2.4)	0 (0)	2 (11.8)	**0**.**038**
Diabetes	14 (16.5)	10 (14.7)	4 (23.5)	0.465
Valvulopathy^a^	4 (4.7)	3 (4.4)	1 (5.9)	1.000
Venous thromboembolism	2 (2.4)	1 (1.5)	1 (5.9)	0.362
Arrhythmias	10 (11.8)	6 (8.8)	4 (23.5)	0.107
Hyperlipidemia	31 (36.5)	26 (38.2)	5 (29.4)	0.499
Hypertension	42 (49.4)	29 (42.7)	13 (76.5)	**0**.**013**
Pacemaker/AICD	2 (2.4)	2 (2.9)	0 (0)	1.000
Cirrhosis	1 (1.2)	1 (1.5)	0 (0)	1.000
Prior cancer	10 (11.8)	10 (14.7)	0 (0)	0.200
NSCLC clinical stage				
Stage I–III	15 (17.9)	11 (16.4)	4 (23.5)	0.492
Stage IV	69 (82.1)	56 (83.6)	13 (76.5)	
Cancer treatments				
Cytotoxic chemotherapy	45 (54.9)	37 (56.9)	8 (47.1)	0.467
Other EGFR inhibitors	20 (25.3)	18 (28.1)	2 (13.3)	0.331
Immunotherapy	8 (10.5)	8 (13.1)	0 (0)	0.334
Thoracic radiotherapy	26 (32.5)	21 (32.3)	5 (33.3)	1.000
CV medications				
Beta-blocker	25 (29.4)	15 (22.1)	10 (58.8)	**0**.**003**
Calcium channel blocker	15 (17.7)	12 (17.7)	3 (17.7)	1.000
ACE inhibitor	12 (14.1)	11 (16.2)	1 (5.9)	0.445
Angiotensin receptor blocker	10 (11.8)	8 (11.8)	2 (11.8)	1.000
ARNI	1 (1.2)	0 (0)	1 (5.9)	0.200
MRA	4 (4.7)	2 (2.9)	2 (11.8)	0.177
Loop diuretic	7 (8.2)	4 (5.9)	3 (17.7)	0.139
Statin	36 (42.4)	30 (44.1)	6 (35.3)	0.510
Baseline lab values				
Creatinine (mg/dl)	0.8 (0.7–1)	0.8 (0.7–0.9)	0.93 (0.8–1.3)	0.061
Hemoglobin A1c (%)	5.7 (5.3–6.4)	5.65 (5.4–6.4)	5.9 (5.28–6.2)	1.000
Total cholesterol	181.77 (±49.0)	178.17 (±50.3)	193.78 (±45.1)	0.409
Triglycerides	96 (71–150)	95 (71–137)	121 (71–195)	0.372
Low-density lipoprotein	95.07 (±40.0)	94.36 (±41.4)	97.33 (±37.6)	0.849
High-density lipoprotein	53 (44–69)	51.5 (45.5–66.3)	60 (34–101)	0.859

Data are presented as number of patients (column%), mean (± standard deviation), or median (IQR, interquartile range).

*P*-value is calculated by analysis of variance or Wilcoxon rank-sum test for continuous variables; and chi-square test or Fisher's exact test for categorical variables as appropriate.

Bold values signifies *p* < 0.05.

HFrEF, heart failure with reduced ejection fraction; AICD, automatic implantable cardioverter defibrillator; NSCLC, non-small cell lung cancer; EGFR, epidermal growth factor receptor; CCB, calcium channel blocker; ACE, angiotensin-converting enzyme; ARNI, angiotensin receptor/neprilysin inhibitor; MRA, mineralocorticoid receptor antagonist.

^a^ Valvulopathy classified as moderate or greater stenosis or regurgitation of the mitral, tricuspid, or aortic valves.

Baseline characteristics were compared between patients who experienced any grade ≥2 cardiac CTCAE (*n* = 17) vs. those who did not (*n* = 68; Table 1). Patients with cardiac events were more likely to have chronic kidney disease (CKD) (11.8% vs. 0%; *p* = 0.038), hypertension (76.5% vs. 42.7%; *p* = 0.013), and be on a beta-blocker (58.8% vs. 22.1%; *p* = 0.003) compared to those who did not experience cardiac events. No significant differences were observed between groups in the usage of other CV medications, including statins, angiotensin-converting enzyme (ACE) inhibitors, and mineralocorticoids (*p* > 0.05).

### Analysis of cardiac events

**Figure 1:**
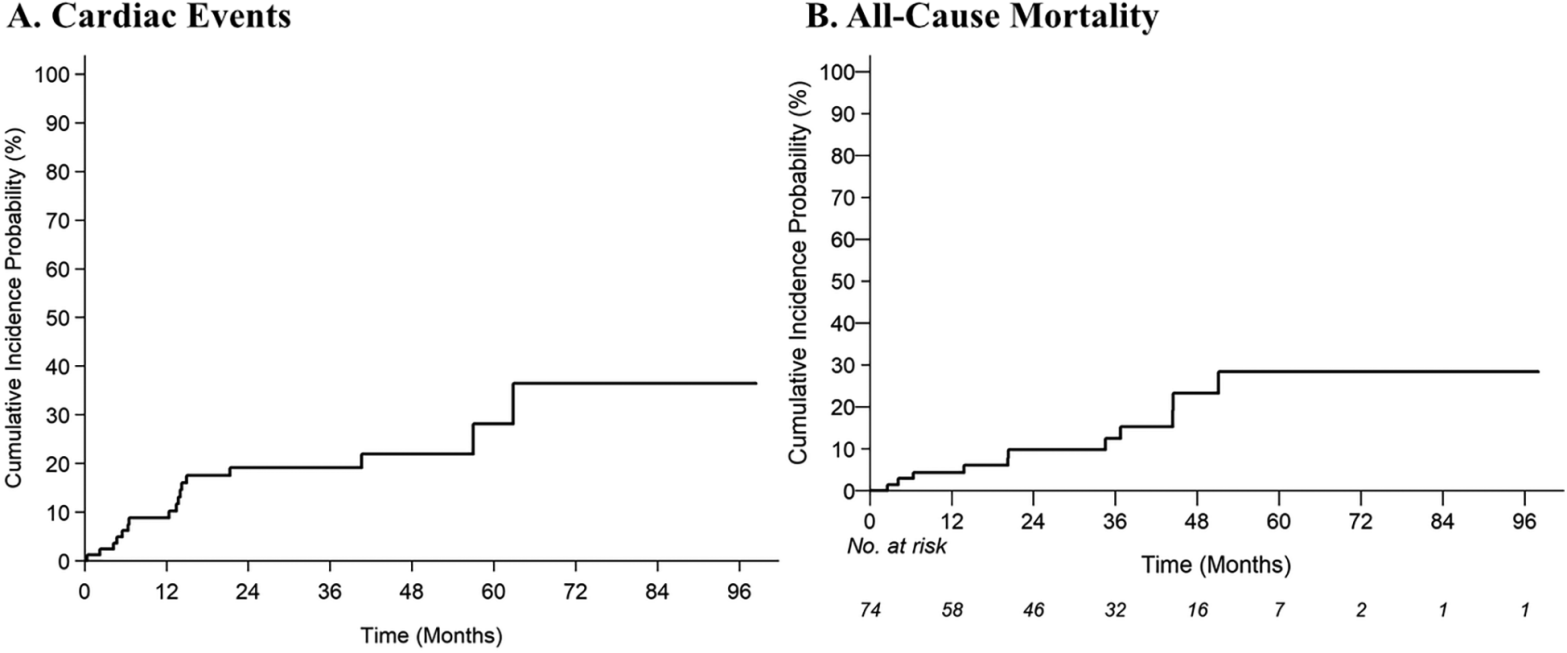
Cumulative incidence estimates of **(A)** grade ≥2 cardiac common terminology criteria for adverse events (CTCAE), and **(B)** all-cause mortality.

With a median follow-up of 34.7 months (95% CI: 26.2–41.5 months), 17 of 85 patients (20.0%) experienced one or more grade ≥2 cardiac CTCAE with a median time to first event of 24.2 months (IQR: 10.8–42.7 months). The 2-year cumulative incidences of grade ≥2 and grade ≥3 cardiac CTCAE were 19.2% (95% CI: 11%–29%) and 8.5% (95% CI: 3.4%–16.5%), respectively (Figure 1). There were 27 grade ≥2 cardiac CTCAE, including QTc prolongation (*n* = 10), EF decline from baseline (of ≥5% if symptomatic or ≥10% if asymptomatic) to ≤50% (*n* = 5), new-onset moderate-to-severe valvular regurgitation or stenosis (*n* = 5), supraventricular tachycardia requiring medication prescription or hospitalization (SVT, *n* = 3), and pericardial tamponade (*n* = 1). Eight patients experienced severe toxicities with six grade 3 [*n* = 3 QTc prolongation (≥501 ms), *n* = 2 SVT, *n* = 1 EF decline to 20%–39% with ≥20% decrease from baseline], and two grade 4 (*n* = 1 EF decline to <20%, *n* = 1 pericardial tamponade) CTCAE. To note, the patient with pericardial tamponade did not have fluid cytology analyzed and had Stage IV disease—thus cancer-related effusion was a possible etiology. There were no cardiovascular deaths/grade 5 cardiac CTCAE. On multivariable analysis, there was an increased risk of grade ≥2 cardiac CTCAE with pre-existing arrythmia [subdistribution hazard ratio (SHR) 3.9; 95% CI: 1.1–13.7; *p* = 0.034] and BMI (SHR 1.07/unit; 95% CI: 1.00–1.14; *p* = 0.036) (Table 2). There was no impact of race/ethnicity on the risk of cardiac events (*p* > 0.05).

**Table 2:** Competing risks and Cox regression analyses for cardiac events and All-cause mortality.

	Grade ≥2 cardiac events					All-cause mortality				
Variable		Univariate		Multivariable			Univariate		Multivariable	
	*n*	HR (95% CI)	*P*-value	HR (95% CI)	*P*-value	*n*	HR (95% CI)	*P*-value	HR (95% CI)	*P*-value
Age, years^a^	85	1.03 (0.97–1.10)	0.295	^b^		74	1.00 (0.94–1.06)	0.889	^b^	
Sex				^b^					^b^	
Male	28	1.37 (0.50–3.72)	0.537			26	0.46 (0.10–2.14)	0.324		
Female	57	1 (Reference)				48	1 (Reference)			
Race/Ethnicity				^b^						
Non-Hispanic Other	7	2.84 (0.82–9.80)	0.099							
Non-Hispanic Asian	19	1.13 (0.31–4.06)	0.853							
Hispanic	13	1.59 (0.41–6.07)	0.500							
Non-Hispanic White	44	1 (Reference)								
BMI^a^	84	1.06 (1.00–1.13)	0.063	1.07 (1.00–1.14)	**0.036**	73	0.99 (0.89–1.10)	0.829	^b^	
Smoking (combined)				^b^					^b^	
Ever smoker	34	0.78 (0.29–2.08)	0.624			30	0.35 (0.07–1.64)	0.181		
Non-smoker	50	1 (Reference)				43	1 (Reference)			
Prior cancer	10	0.00 (0.00-NA)	**<0**.**001**	^b^		10	0.55 (0.07–4.33)	0.569	^b^	
NSCLC clinical stage				^b^					^b^	
Stage I/II/III	15	1.69 (0.58–4.94)	0.338			15	0.46 (0.06–3.57)	0.454		
Stage IV	69	1 (Reference)				58	1 (Reference)			
Prior chemotherapy	45	0.54 (0.21–1.41)	0.209	^b^		36	1.34 (0.33–5.43)	0.681	^b^	
Prior EGFR inhibitors	20	0.22 (0.05–0.95)	**0**.**042**	^b^		16	4.82 (1.19–19.48)	**0**.**027**	6.37 (1.48–27.32)	**0**.**013**
Prior immunotherapy	8	0.00 (0.00–0.00)	**<0**.**001**	^b^		5	3.04 (0.63–14.69)	0.167	^b^	
Prior thoracic radiation	26	0.91 (0.33–2.51)	0.848	^b^		22	0.96 (0.24–3.86)	0.959	^b^	
Baseline CV risk factors										
CHD	10	1.10 (0.22–5.41)	0.904	^b^		7	1.20 (0.15–9.50)	0.860	^b^	
Heart failure	1	12.62 (5.70–27.94)	**<0**.**001**	Not considered		0	NA		Not considered	
CKD	2	15.19 (4.63–49.82)	**<0**.**001**	^b^		2	6.49 (0.78–54.24)	0.084	^b^	
Arrhythmias	10	4.21 (1.38–12.87)	**0**.**012**	3.90 (1.11–13.72)	**0.034**	9	4.77 (0.93–24.58)	0.062	^b^	
Hypertension	42	4.25 (1.45–12.46)	**0**.**008**	^b^		38	0.35 (0.09–1.33)	0.122	^b^	
Pacemaker or AICD	2	0.00 (0.00-NA)	**<0**.**001**			1	68.45 (4.28–1,094.92)	**0**.**003**	^b^	
Cirrhosis	1	0.00 (0.00-NA)	**<0**.**001**	^b^		1	0.00 (0.00–NA)	0.995	^b^	
CV medications										
Beta-blocker	25	4.06 (1.58–10.42)	**0**.**004**	^b^		21	0.00 (0.00–NA)	0.993	^b^	
CCB	15	1.29 (0.35–4.68)	0.703	^b^					^b^	
ACE inhibitor	12	0.38 (0.05–2.84)	0.346	^b^		11	1.21 (0.26–5.61)	0.811	^b^	
ARB	10	0.94 (0.21–4.11)	0.933	^b^		9	0.00 (0.00-NA)	0.995	^b^	
ARNI	1	12.62 (5.70–27.94)	**<0**.**001**	^b^		0	NA		^b^	
MRA	4	3.82 (1.08–13.54)	**0**.**038**	^b^		4	2.16 (0.28–16.96)	0.463	^b^	
Loop diuretic	7	3.71 (1.00–13.79)	0.050	^b^		5	5.26 (1.11–24.98)	**0**.**037**	9.97 (1.75–56.82)	**0**.**010**
Statin	36	0.69 (0.26–1.80)	0.445	^b^		31	0.20 (0.04–0.97)	**0**.**046**	^b^	
Baseline CV labs										
Creatinine (mg/dl)^a^	69	3.06 (0.72–12.95)	0.129	^b^		62	0.67 (0.03–16.12)	0.807	^b^	
A1c (%)^a^	31	1.18 (0.63–2.21)	0.603	^b^		29	0.58 (0.10–3.23)	0.530	^b^	
Total cholesterol^a^	39	1.01 (1.00–1.02)	0.169	^b^		36	0.99 (0.96–1.02)	0.319	^b^	
Triglycerides^a^	38	1.01 (1.00–1.01)	0.108	^b^		36	0.99 (0.96–1.02)	0.421	^b^	
LDL^a^	38	1.00 (0.99–1.02)	0.531	^b^		36	0.98 (0.95–1.02)	0.282	^b^	
HDL^a^	37	1.01 (0.99–1.04)	0.343	^b^		35	1.01 (0.97–1.05)	0.677	^b^	

A total of 84 observations were used in the multivariable model for cardiac events [grade ≥2 Common Terminology Criteria for Adverse Events (CTCAE)] and 70 observations were used in the multivariable model for all-cause mortality. There are categorical variables with few patients or outcome events in some categories. Results with no outcome events (e.g., SHR: 0.00; 95% CI: 0.00—NA) were not interpreted as statistically significant as it may be due to small sample sizes.

Bold values signifies *p* < 0.05.

SHR, subdistribution hazard ratio; HR, hazard ratio; CI, confidence interval; BMI, body mass index; CHD, coronary heart disease; HFrEF, heart failure with reduced ejection fraction; CKD, chronic kidney disease; VTE, venous thromboembolism; AICD, automatic implantable cardioverter defibrillator; NSCLC, non-small cell lung cancer; EGFR, epidermal growth factor receptor; CCB, calcium channel blocker; ACE, angiotensin-converting enzyme; ARB, angiotensin receptor blocker; ARNI, angiotensin receptor/neprilysin inhibitor; MRA, mineralocorticoid receptor antagonist; LDL, low-density lipoprotein; HDL, high-density lipoprotein.

^a^ Hazard ratio is expressed as 1-unit increment.

^b^ Dropped out of the model.

### Analysis of all-cause mortality

With a median follow-up of 34.7 months, 13% of patients died (8% of lung cancer and 5% of known non-cardiac cause). The 2-year estimate of all-cause mortality was 9.8% (95% CI: 3.91–18.98). On multivariable analysis, prior EGFR inhibitors (HR 6.37; 95% CI: 1.48–27.32; *p* = 0.013) and use of loop diuretics (HR 9.97; 95% CI: 1.75–56.82; *p* = 0.010) were associated with an increased risk of all-cause mortality (Table 2).

### Dynamic electrocardiogram and echocardiographic changes

There were 64 patients with ECGs prior to osimertinib and 73 patients with ECGs after osimertinib initiation (Table 3). The most common ECG change that occurred with Osimertinib treatment was QTc prolongation. The average QTc length was significantly prolonged from 437 (IQR, 422–450 ms) to 451 (IQR, 432–474) ms after osimertinib administration (*p* < 0.001). Grade 3 QTc prolongation (≥501 ms) occurred in three patients, grade 2 QTc prolongation (481–500 ms) occurred in seven patients, and grade 1 QTc prolongation (450–480 ms) occurred in 18 patients. No statistically significance changes were observed in other ECG parameters, including rhythm changes or conduction abnormalities.

**Table 3:** Electrocardiogram and echocardiogram characteristics at baseline and post-osimertinib therapy.

Electrocardiogram characteristics	Baseline ECG (*n* = 64)	Post-osimertinib ECG (*n* = 73)	*P*-value^c^
HR, beats/min, mean (±SD)	76.3 (±12.6)	82.4 (±15.5)	**0** **.** **029**
PR length, median (IQR)	158 (138–174)	148 (134–168)	0.094
1st degree AV block	4 (6.9)	3 (4.4)	0.317
QTc length, median (IQR)	436.5 (421.5–449.5)	451 (432–474)	**<0**.**001**
Interventricular block	7 (10.9)	7 (10.6)	0.480
LVH	5 (7.8)	3 (4.1)	0.564
P wave abnormality^a^	7 (10.9)	4 (5.5)	0.414
Low QRS voltage	3 (4.7)	7 (9.7)	0.317
Pathological Q waves	5 (7.9)	6 (8.2)	NA^b^
Normal sinus rhythm	52 (81.3)	63 (86.3)	0.206
Sinus tachycardia	3 (4.7)	5 (6.9)	1.000
Sinus bradycardia	3 (4.7)	1 (1.4)	0.083
Atrial fibrillation/flutter	5 (7.8)	4 (5.5)	NA^b^
PACs/PVCs	5 (9.8)	6 (8.3)	0.317
Echocardiogram characteristics	Baseline TTE (*n* = 38)	Post-osimertinib TTE (*n* = 85)	*P*-value^c^
LVEF, mean (±SD)	60.8 (±8.5)	57.54 (±11.5)	**0**.**002**
LVEF (≤50%)	2 (5.3)	9 (10.6)	**0**.**046**
Diastolic dysfunction			
Mild	12 (31.6)	34 (40.0)	0.532
Moderate/severe	6 (15.8)	6 (7.1)	1.000
LVIDd, mean (±SD)	4.4 (±0.6)	4.36 (±0.7)	0.082
IVSd, median (IQR)	1 (0.81–1.2)	1 (0.81–1.2)	0.563
LVPWd, mean (±SD)	1.0 (±0.2)	1 (±0.3)	0.792
LA area, mean (±SD)	19.2 (±6.8)	16.1 (±5.4)	0.843
LA volume index, mean (±SD)	28.9 (±14.0)	24.9 (±12.4)	**0**.**036**
TAPSE, mean (±SD)	2.1 (±0.3)	2.0 (±0.4)	0.916
TR peak velocity, mean (±SD)	237.7 (±72.8)	NA	NA
PASP, mean (±SD)	33.1 (±13.9)	28.1 (±11.2)	**0**.**034**
Mitral stenosis	0	0	NA
Aortic stenosis			
Mild	2 (5.3)	1 (1.2)	1.000
Moderate	1 (2.6)	1 (1.2)	
Mitral regurgitation			
Mild	10 (26.3)	29 (34.1)	0.317
Moderate	1 (2.6)	3 (3.5)	1.000
Severe	1 (2.6)	0 (0)	NA
Aortic regurgitation			
Mild	4 (10.5)	12 (14.1)	0.157
Moderate	2 (5.3)	3 (3.5)	

Data are presented as number of patients (column%), mean (±SD), or median (IQR, interquartile range).

*P*-value is calculated by a paired *t*-test or signed rank test for continuous variables, and McNemar's test for categorical variables.

Bold values signifies *p* < 0.05.

^a^ Suggestive of left atrial enlargement.

^b^ There are no discordant pairs.

^c^ Statistical analysis was done in patients with both baseline and post-Osimertinib electrocardiogram and echocardiogram.

Among 85 patients with at least one echocardiogram after osimertinib initiation, 38 had at least one echocardiogram prior to treatment (Table 3). The median time between the start of osimertinib and follow-up echocardiogram completion was 17.6 months (95% CI: 13.7–21.4). When comparing post- vs. pre-osimertinib echocardiographic variables, there was a significant decline in LVEF from 60.8 ± 8% vs. 57.5 ± 11% (*p* = 0.002), and in particular—LVEF ≤50% was observed with increased frequency following osimertinib treatment (10.6% vs. 5.3% of patients; *p* = .046). Grade 4 LV dysfunction (LVEF reduction to <20%) occurred in one patient, grade 3 LV dysfunction (LVEF decline to 20%–39% with ≥20% decrease from baseline) occurred in one patient, and grade 2 LV dysfunction (LVEF decline by ≥10% from baseline to 40%–50%) occurred in three patients. The five patients with osimertinib-associated left ventricular dysfunction are described Table 4. Of these five patients, three had some degree of recovery but had persistently low LVEF (case #1, 2, and 4), while one patient had worsening of LVEF despite discontinuation of osimertinib (case #5), and one patient was lost to follow-up (case #3). In addition, post-osimertinib left atrial (LA) area was significantly increased in patients with cardiac events vs. those without cardiac events (18.8 ± 5.0 cm^2^ vs. 15.3 ± 5.3 cm^2^, *p* = 0.029, Supplementary Table 1). Grade 2 valvular disease occurred in five (6%) patients in the cohort: three patients developed moderate aortic regurgitation, one developed moderate mitral regurgitation, and one developed moderate tricuspid regurgitation. No significant differences were observed in other echocardiographic parameters.

**Table 4:** Characteristics of patients with osimertinib-associated left ventricular dysfunction.

	Case 1	Case 2	Case 3	Case 4	Case 5
Age, years	75	81	75	81	48
Sex	Female	Female	Female	Male	Female
EGFR mutation	Exon 19 deletion	Exon 19 deletion	Unknown	Unknown	L858R
Osimertinib line	2nd	1st	1st	1st	2nd
RT, total Gy	50	–	-	–	38
Tobacco use	No	No	No	Former	No
CVD history	None	HTN, DM, CKD, Atrial fibrillation	HTN	CAD, HTN, DM, HLD	None
CTCAE grade	2	3	4	3	2
Time to event	41 months	5 months	6 months	6 months	13 months
MACE	No	No	Yes; HF admission	Yes; HF admission	No
Pre-osimertinib LVEF,%	60	50	Unknown	50	64
Post-osimertinib LVEF,%	40	33	15	20	44
Osimertinib treatment adjustments	Reduced from 80 mg–40 mg for 2 years and then increased back to 80 mg	Temporarily held and resumed at half dose	discontinued	Temporarily held	Discontinued
HF treatment	Carvedilol 3.125 mg; Losartan 25 mg; Spironolactone 25 mg	Carvedilol 3.125 mg; Dapagliflozin 10 mg	Sacubitril-valsartan 24–26 mg; Carvedilol 3.125 mg; Furosemide 20 mg	Valsartan 40 mg	None
LVEF improvement	47% after 19 months	45% after 4 months	NA; lost to follow-up	37% after 27 months	40% after 9 months

EGFR, epidermal growth factor receptor; RT, radiation therapy; CVD, cardiovascular disease; HTN, hypertension; DM, diabetes mellitus; CKD, chronic kidney disease; CAD, coronary artery disease; HLD, hyperlipidemia; CTCAE, common terminology criteria for adverse events; MACE, major adverse cardiovascular event; LVEF, left ventricular ejection fraction; HF, heart failure.

## Discussion

In this retrospective single-center study of 85 patients with NSCLC treated with osimertinib and who received at least one echocardiogram following osimertinib initiation, we made the following observations: (1) this patient cohort is at very high risk of CV events with 2-year cumulative incidence of grade ≥2 CV CTCAE of 19% and grade ≥3 CV CTCAE of 8.5%; (2) The most common CV adverse events with osimertinib therapy were QTc prolongation, LV dysfunction (with or without heart failure symptoms), progressive valvular disease, and SVTs; (3) there appears to be a latency in the manifestation of CV events with a median time to first event of 24.2 months (IQR: 10.8–42.7 months); and (4) pre-existing arrythmia and BMI were identified as risk factors for cardiac events following osimertinib treatment. A strength of this study is the availability of serial electrocardiogram and echocardiographic analysis in one of the largest NSCLC cohorts that builds upon recent studies (14, 26, 27) Additionally, BMI was identified as a modifiable risk factor for osimertinib-associated CV toxicity, and validated in further studies, represents an opportunity for risk mitigation in patients who receive osimertinib.

Several issues warrant further discussion. First, our observation of a nearly 20% 2-year cumulative incidence of grade ≥2 cardiac CTCAE rate is higher than several recent reports. A single-center retrospective cohort study from Japan reported 5% incidence of grade 3 or higher CTCAE (26). In the prior study, the number of patients who had echocardiograms was small (*n* = 36) and the baseline cardiovascular risk profile vastly differed from the US population, where cardiovascular co-morbidities are significantly more prevalent. Indeed, our study observed a higher prevalence of hypertension, diabetes, and prior/current smoking use in the US cohort, which might have contributed to the increased rate of osimertinib-associated cardiotoxicity. Further, a retrospective analysis on the FDA Adverse Events Reporting System (FAERS), a pharmacovigilance database, showed that osimertinib (vs. standard therapies) was associated with higher rates of grade ≥3 cardiac events such as QTc prolongation, cardiac failure, a decline in left ventricular ejection fraction (LVEF), among others (14). However, this study has several limitations, including heterogeneity in individual reporting and details of the adverse events (e.g., grade of toxicity), as well as unknown baseline characteristics. In addition, because FAERS is a voluntary reporting system, it likely is an inaccurate estimate of the true incidence of cardiac events.

LV dysfunction is well-described toxicity of osimertinib therapy and is similarly observed in the current study with incidence of 6% among those patients who have had echocardiogram performed post-osimertinib initiation (12–14.) The characterization of the true prevalence of LV dysfunction in osimertinib treated patients is limited by the inconsistent echocardiographic monitoring practices across centers. A small single-center series of 17 patients with LV dysfunction suspected to be causally linked to osimertinib demonstrated that only in half of the cases was echocardiogram performed as part of screening strategy and the majority of echocaridograms were performed due to symptoms (28). Similalry, this inconsistent echocardiographic monitoring limits our understanding of the timing of onset of toxicities, so that monitoring is implemented at the most vulnerable window. In our cohort of patients, of the five cases of LV dysfunction observed, three had some degree of improvement in their LVEF on subsequent echocardiograms after initiation of guideline-directed medical therapy (Table 4).

Similar to other reports (12–14), the most common ECG change observed in the current study following osimertinib therapy was QTc prolongation (median 14 ms). Importantly, however, clinically significant polymorphic ventricular arrhythmias are infrequently witnessed until the QTc interval exceeds 500 ms (29). The majority of observed events (25/28) were grade 1 (450–480 ms) and 2 (481–500 ms), while three patients developed grade 3 QTc prolongation (>500 ms)—though there were no episodes of torsade de pointes, polymorphic ventricular tachycardia, or other clinical consequence of QTc prolongation. However, several case reports have described osimertinib-induced ventricular arrhythmias (30–32.) Thus, regular clinical monitoring should be performed, including ECG surveillance—particularly if other QTc prolonging drugs are used (anti-emetics, antibiotics), correcting electrolyte imbalances, and discontinuing other unnecessary QTc prolongaing medications. Further, temporary drug interruption and resumption at a lower daily dose is recommended in patients who develop grade 3 QTc prolongation (33).

While the precise mechanism of osimertinib-associated cardiotoxicity has yet to be elucidated, *in vitro* studies have shown that osimertinib can inhibit human epidermal growth factor receptor 2 (HER2) (34), which is important in maintaining cardiac function (35). Given that anti-HER2 therapies (such as trastuzumab) are also associated with cardiac toxicity (36), this suggests that off target effects may potentially contribute to the enhanced toxicity profile of this drug. Further, given the increasing use of osimertinib in the curative setting, there is a growing need for improved baseline CV risk stratification, early cardiac event detection, and adequate surveillance of cardiotoxicity. In fact, the comparable rates of LV dysfunction observed with osimertinib vs. HER2 antagonists (37–41) calls for diligent echocaridograhic monitoring in osimertinib-treated patients with protocols that mirror those of HER2 therapies. In recognition of this toxicity, the European Society of Cardiology Cardio-Oncology guidelines recommend baseline echocardiogram in patients prior to starting osimertinib (Class I, Level B evidence) and consideration of performing echocardiograms every 3 months while patients are maintained on osimertinib (Class IIA, Level B evidence). Our studies and others are a call for action in the oncology and cardio-oncology community—namely, for a call for more systematic monitoring for LV dysfunction, valvular disease progression, and QTc prolongation for patients on osimertinib therapy, particularly as we enter an era of increased osimertinib use as curative-intent therapy in patients with non-metastatic disease.

This study has several limitations. Given its retrospective nature, this could under- (or over-) estimate the true incidence of cardiac dysfunction, including in patients with limited follow-up due to competing risk or medical care received locally. In addition, being a tertiary referral center where more medically complex patients are evaluated, could also over-estimate the incidence of cardiac dysfunction. Further, this cohort is a subset that underwent echocardiograms, which may impart a selection bias as these patients may be at higher CV risk than those that did not, particularly in the retrospective setting. There were fewer echocardiograms available prior to osimertinib compared to during treatment, which may lead to overestimation of changes in echocardiographic metrics. Similarly, given the variable timing of echocardiograms across the cohort, the most recent study was analyzed—though this may not fully capture dynamic changes over time. Additionally, while prior thoracic radiotherapy was not associated with cardiac event risk in this study, the impact of cardiac substructure radiation dose exposure could be a contriobuting factor (42), which is not fully captured here given the small numbers of patients treated with RT and small event numbers. Similarly, given the high baseline cardiovascular risk and exposure to multiple potential cardio-toxic cancer therapies, the observed cardiac events may not be solely attributed to Osimertinib and may reflect several contributing risk factors. Further, given the limited sample size, the impact of concurrent cardiovascular medical therapies could not be fully assessed. Lastly, as this was a sample size of convenience, the treatment years spanned 15 years and may not be fully generalizable to a modern treatment cohort.

Together, these findings underscore the importance of early referral to cardiology or cardio-oncology for patients who develop osimertinib-associated cardiotoxicity. Additionally, in our study there was a significant increase in LA area and a trend toward increased left atrial volume index (LAVI) post-osimertinib among patients with cardiac events (vs. no cardiac events) (Supplementary Table 1). These remodeling changes may predispose patients to developing arrhythmias, such as atrial fibrillation—which has been associated with the osimertinib cardiac toxicity spectrum (14). Longitudinal studies of longer duration will be needed to characterize the long-term sequelae of treatment with Osimertinib.

## Conclusion

In this retrospective cohort study, cardiac events were common after Osimertinib treatment, with nearly a 20% 2-year cumulative incidence of grade ≥2 cardiac CTCAE and 9% 2-year cumulative incidence of grade ≥3 cardiac CTCAE. Importantly, several electrocardiogram and echocardiographic changes were observed, including QTc prolongation and reduced LVEF. BMI was identified as a potentially modifiable risk factor for Osimertinib-associated cardiac events, worthy of further study. These findings highlight the need for optimized risk mitigation approaches following Osimertinib treatment, including identifying patients who might benefit from intensified surveillance.

## Data Availability

The original contributions presented in the study are included in the article/Supplementary Material, further inquiries can be directed to the corresponding author.
