# Supplementary material for: Cardiac events and dynamic echocardiographic and electrocardiogram changes following osimertinib treatment in lung cancer
Source: Front Cardiovasc Med. 2024 Dec 16;11:1485033. doi: 10.3389/fcvm.2024.1485033 (PMC11685755; doi:10.3389/fcvm.2024.1485033)
Supplement: Supplementary file 1 [file Datasheet1.pdf]

Supplementary Figure 1. CONSORT diagram depicting included patients.

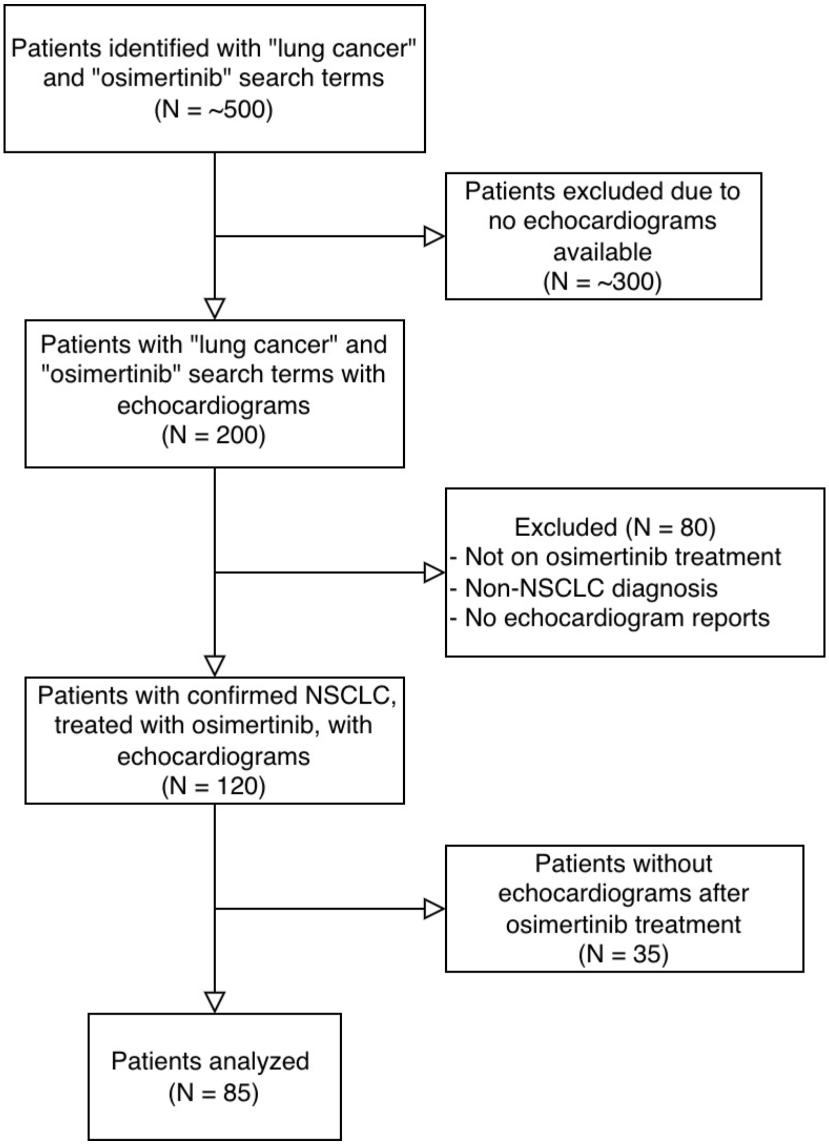

Supplementary Table 1. Electrocardiogram and echocardiogram characteristics post-osimertinib therapy in patients with and without cardiac events.

**Electrocardiogram characteristics**	**Cardiac Events (n = 17)**	**No Cardiac Events (n = 68)**	**P-value**
HR, beats/min, mean (± SD)	84.6 (± 14.0)	81.7 (± 16.0)	0.509
PR length, median (IQR)	143 (126 - 184)	148 (138 - 166)	0.668
1st degree AV block	1 (6.7)	2 (3.8)	0.533
QTc length, mean (± SD)	478 (± 33.1)	446.4 (± 24.5)	**<0.001**
Interventricular block	2 (16.7)	5 (9.3)	0.602
LVH	1 (5.9)	2 (3.6)	0.554
P wave abnormality[#]	1 (5.9)	3 (5.4)	1.000
Low QRS voltage	1 (5.9)	6 (10.9)	1.000
Pathological Q waves	4 (23.5)	2 (3.6)	**0.024**
Normal sinus rhythm	14 (82.4)	49 (87.5)	0.689
Sinus tachycardia	1 (5.9)	4 (7.1)	1.000
Sinus bradycardia	0 (0)	1 (1.8)	1.000
Atrial fibrillation/flutter	2 (11.8)	2 (3.6)	0.230
PACs/PVCs	3 (17.7)	3 (5.5)	0.139
**Echocardiogram characteristics**	**Cardiac Events (n = 17)**	**No Cardiac Events (n = 68)**	**P-value†**
LVEF, median (IQR)	55 (40 - 60)	60 (56 - 64)	**0.024**
LVEF (≤ 50%)	5 (29.4)	4 (5.9)	**0.014**
Diastolic dysfunction			
Mild	2 (33)	9 (35)	0.166
Moderate/Severe	2 (33)	1 (4)	
LVIDd, median (IQR)	4.7 (4.3 - 5.1)	4.3 (3.8 - 4.8)	**0.043**
IVSd, mean (± SD)	1.05 (± 0.3)	0.99 (± 0.2)	0.364
LVPWd, median (IQR)	0.98 (0.87 - 1.1)	1 (0.83 - 1.1)	0.821
LA area, mean (± SD)	18.81 (± 5.0)	15.33 (± 5.3)	**0.029**
LA volume index, median (IQR)	33.55 (19.7 - 39)	20.3 (15.8 - 31.2)	0.102
TAPSE, mean (± SD)	1.87 (± 0.4)	2.09 (± 0.4)	0.106
TR peak velocity, mean (± SD)	257.58 (± 64.9)	230.63 (± 44.8)	0.094
PASP, median (IQR)	29.5 (22 - 53)	24.5 (20 - 32)	0.144
Mitral Stenosis	0	0	NA
Aortic Stenosis			
Mild	0 (0)	1 (1.5)	1.000
Moderate	1 (5.9)	0 (0)	
Mitral Regurgitation			
Mild	8 (47)	21 (30.9)	0.224
Moderate	2 (11.8)	1 (1.5)	
Aortic Regurgitation			
Mild	2 (11.8)	10 (14.7)	**0.022**
Moderate	3 (17.6)	0 (0)	

Data are presented as number of patients (column %), mean (± SD), or median (IQR, interquartile range).
P-value is calculated by ANOVA or Wilcoxon rank-sum test for continuous variables;
and chi-square test or Fisher's exact test for categorical variables, where appropriate.
[#]Suggestive of left atrial enlargement.
